# RETRACTED: Haghmorad et al. Oral Administration of Myelin Oligodendrocyte Glycoprotein Attenuates Experimental Autoimmune Encephalomyelitis through Induction of Th2/Treg Cells and Suppression of Th1/Th17 Immune Responses. *Curr. Issues Mol. Biol.* 2022, *44*, 5728–5740

**DOI:** 10.3390/cimb47090781

**Published:** 2025-09-20

**Authors:** Dariush Haghmorad, Bahman Yousefi, Majid Eslami, Ali Rashidy-Pour, Mahdieh Tarahomi, Maryam Jadid Tavaf, Azita Soltanmohammadi, Simin Zargarani, Aleksandr Kamyshnyi, Valentyn Oksenych

**Affiliations:** 1 Cancer Research Center, Semnan University of Medical Sciences, Semnan 35131, Iran; dhaghmorad@gmail.com (D.H.); yosefi_bahman@semums.ac.ir (B.Y.); 2 Department of Immunology, Semnan University of Medical Sciences, Semnan 35131, Iran; 3 Department of Bacteriology and Virology, Semnan University of Medical Sciences, Semnan 35131, Iran; m.eslami@semums.ac.ir; 4 Research Center of Physiology, Semnan University of Medical Sciences, Semnan 35131, Iran; rashidy-pour@semums.ac.ir; 5 Department of Microbiology, Virology and Immunology, I. Horbachevsky Ternopil National Medical University, 46001 Ternopil, Ukraine; 6 Institute of Clinical Medicine, University of Oslo, 0318 Oslo, Norway

The journal retracts the article “Oral Administration of Myelin Oligodendrocyte Glycoprotein Attenuates Experimental Autoimmune Encephalomyelitis through Induction of Th2/Treg Cells and Suppression of Th1/Th17 Immune Responses” [1] cited above.

Following publication, concerns were brought to the attention of the Editorial Office regarding the integrity of some images presented in this publication [1].

Adhering to our standard procedure, an investigation was conducted by the Editorial Office and Editorial Board that identified indications of inappropriate image reuse between figures presented in this article [1] and earlier publications [2,3], produced by the same research group and presenting different experimental settings. While the authors collaborated within this process, raw material meeting the journal’s original images requirements (https://www.mdpi.com/journal/cimb/instructions#oriimages, accessed 13 August 2025) could not be provided for Editorial Board evaluation. Consequently, the Editorial Board has lost confidence in the reliability of the findings and has decided to retract this publication, as per MDPI’s retraction policy (https://www.mdpi.com/ethics#_bookmark30).

This retraction was approved by the Editor-in-Chief of the *CIMB* journal.

Author Dariush Haghmorad agreed with this retraction. The remaining authors stated that they were unaware of the image reuse, cooperated fully with the investigation, and agreed with the decision to retract the article.
